# Point-of-Care Ultrasound May Reduce Misdiagnosis of Pediatric Intussusception

**DOI:** 10.3389/fped.2021.601492

**Published:** 2021-02-04

**Authors:** Hsiang-Ju Hsiao, Chao-Jan Wang, Chien-Chung Lee, Yi-Chen Hsin, Sze-Yuen Yau, Shih-Yen Chen, Wan-Chak Lo, Patricia-Wanping Wu, Chyi-Liang Chen, Yi-Jung Chang

**Affiliations:** ^1^Department of Pediatrics, Chang Gung Memorial Hospital, Chang Gung University College of Medicine, Taoyuan, Taiwan; ^2^Department of Pediatrics, Chang Gung Memorial Hospital, Keelung, Taiwan; ^3^Department of Radiology, Chang Gung Memorial Hospital, Chang Gung University College of Medicine, Taoyuan, Taiwan; ^4^Molecular Infectious Disease Research Center, Chang Gung Memorial Hospital, Taoyuan, Taiwan; ^5^Chang Gung Medical Education Research Centre, Chang Gung Memorial Hospital, Linkou, Taiwan; ^6^Department of Pediatrics, Taipei Medical University Shuang Ho Hospital, New Taipei City, Taiwan

**Keywords:** intussusception, point-of-care system, ultrasonography, child, diagnosis

## Abstract

**Aim:** Intussusception, the most common abdominal emergency in early childhood, is frequently misdiagnosed at initial presentation. The effect of using point-of-care ultrasonography (POCUS) by emergency medicine physicians on pediatric intussusception misdiagnosis rate remains unclear. Here, we summarize outcomes and misdiagnoses before and after training junior and senior physicians on using POCUS for diagnosing intussusception and compared their performance levels.

**Materials and Methods:** This observational cohort analysis included patients with suspected intussusception who visited a pediatric emergency department (ED) between January 2017 and December 2019. All enrolled patients were evaluated by junior (<10-year experience) or senior attending physicians. Misdiagnosis was defined as a finding of negative air reduction or confirmation of diagnosis on ED revisit or admission. The misdiagnosis rates and outcomes before and after POCUS training for intussusception diagnosis were evaluated and performance of the junior and senior physicians was compared.

**Results:** Of the 167 enrolled patients, 130 were confirmed to have intussusception by air reduction. Misdiagnosis rate was significantly lower in the post-training patient group after training than in the pre-training patient group (43.7 vs. 12.7%, *P* < 0.001). After training, fewer misdiagnoses were made by the junior (59.1 vs. 25.9%, *P* = 0.003) and senior (31.7 vs. 0%, *P* < 0.001) physicians. In the post-training patient group, the door-to-reduction time and rate of ultrasonography consultation with an expert also decreased significantly (118.2 ± 124.5 vs. 198 ± 250.2 min, *P* = 0.006). Abdominal pain (80.9%) was the most common symptom of intussusception, followed by vomiting (58.3%), fever (17.8%), bloody stool (15.4%), and diarrhea (14.2%). Even after training, the presenting symptoms of intussusception often leading junior physicians to misdiagnosis were diarrhea and fever.

**Conclusions:** A brief POCUS training leads to decreased misdiagnosis rates in both the senior and junior physicians. Junior physicians should increase their awareness regarding diarrhea and fever being the presenting symptoms of intussusception, particularly in early childhood. Combining clinical judgment and POCUS results forms the core principle of the evaluation of children with intussusception.

## Introduction

Intussusception is a common abdominal emergency in children (1–3). Its misdiagnosis and delayed diagnosis in children is common because its symptoms at presentation are highly atypical, mimicking other conditions such as acute gastroenteritis and viral infections (4, 5). The misdiagnosis and delayed treatment of intussusception may lead to severe morbidities including intestinal ischemia, necrosis, perforation, or even mortality (6–8).

To make an accurate clinical diagnosis and confirm the clinical suspicion of intussusception, imaging studies are essential (5). Supine abdominal radiography is typically the first imaging procedure conducted in the emergency department (ED). However, the sensitivity of the interpretation of radiographs by pediatric emergency physicians for intussusception can be as low as 48% (9). Nevertheless, when conducted by experts, ultrasonography (US) is a reliable tool for intussusception diagnosis, with high sensitivity (98%) and specificity (98%) (10–12). Therefore, US is recommended as the diagnostic modality of choice for pediatric intussusception. However, numerous centers do not have 24-h access to US performed by experts (13, 14). The unavailability of US is frequently challenging for pediatric emergency physicians and thus, barium or air enema has been used for further diagnosis and reduction of intussusception over the past decades. Recently, point-of-care US (POCUS) has become the first-line test for emergency physicians to make a timely diagnosis of intussusception at the bedside (13–18). Accordingly, the scenario of imaging studies in the workup of suspicion of intussusception has changed after the introduction of POCUS. Because US is highly operator-dependent, its diagnosis efficacy also depends on the examiner's skills (19–21). When a positive result for intussusception is obtained through POCUS, the enema is reserved for therapeutic purposes. However, if POCUS shows questionable results, the enema may be necessary for both diagnosis and therapy. Moreover, when diagnosis through POCUS is inconclusive, US performed by radiologists, not by pediatric emergency physicians (not skilled in performing US), may be recommended.

At present, the effects of POCUS training pediatric intussusception diagnosis are unclear. Studies on the use of POCUS for intussusception diagnosis by emergency medicine physicians have been limited to performance levels and individual experience levels (22, 23). However, some studies have demonstrated that POCUS may improve the rate of pediatric intussusception misdiagnosis by clinical personnel with varied levels of experience (24, 25). Therefore, we conducted this observational cohort study on the effects of POCUS training on pediatric intussusception misdiagnosis in junior and senior attending physicians. In brief, we evaluated reductions in the rates of misdiagnosis by junior and senior attending physicians after POCUS training and compared the results.

## Materials and Methods

### Study Design Setting

We retrospectively enrolled children who underwent POCUS for intussusception evaluation at a pediatric ED from the end of December 2018 to December 2019. We also retrospectively reviewed the medical records of pediatric ED patients with suspected intussusception from January 2017 to December 2018.

The study was performed at a tertiary care medical center at the largest hospital in Northern Taiwan. The pediatric ED here receives ~36,000 visits annually. An experienced pediatric gastroenterologist performed US at our institution as the gold standard for intussusception diagnosis. Pneumatic enema under fluoroscopic guidance is also used for diagnosis and is the standard of care for the non-surgical reduction of ileocolic intussusception. In Taiwan, pediatric gastroenterologist instead of the radiologist demonstrated the most diagnostic US for pediatric intussusception. Access to US by experts for intussusception evaluation is limited at night and during holidays. During the early study period, no exclusive POCUS training and use were available at our pediatric ED. Physicians performed diagnoses until the end of December 2018 solely on the basis of US conducted by an expert during the daytime or clinical suspicion during the night and holidays. Air enema was further used after obtaining diagnoses using US at daytimes as the therapeutic modality or as the diagnostic and therapeutic choice during night times and holidays after clinical suspicion. After effective air enema, physicians observed children at the hospital for possible recurrence, perforation, and dehydration. If children exhibited peritonitis, pneumoperitoneum, a lead point with a mass lesion, or failed reduction with air enema, surgery was performed. Since the end of December 2018, we implemented POCUS training and use at the pediatric ED. The 8-h pediatric POCUS training included a focused assessment of the heart, aorta, lung, kidney, urinary bladder, and abdomen. Approximately 20–30 min of lecture was dedicated to the abdomen assessment with an image review and at least 40 min of hands-on education. These images showed cases consistent with intussusception or cases with normal bowel. During the hands-on scan training, participants were trained on conducting POCUS use for intussusception under the supervision of pediatric gastroenterologists. This abdomen-focused training session was conducted to enable participants to either rule in or rule out the presence of an ileocolic intussusception. The transducer was placed in the upper right quadrant, where the liver served as the landmark. A complete POCUS scan was followed by the examination of all four quadrants of the abdomen along the colon. The lecture standardized these scanning views by modifying the proposal of intussusception diagnosis in children established in 2012 (25). The relevant institutional review board approved the study.

### Selection of Participants

Children clinically suspected of having intussusception at the pediatric ED or confirmed to have intussusception by means of air enema or US conducted by an expert at the hospital during the study period were enrolled. Data of the enrolled patients were obtained from the medical records diagnoses based on based on the *International Classification of Diseases, Tenth Revision* (ICD-10) code (K56.1) or the electronic record system in the radiology department with diagnoses based on fluoroscopic reduction of intussusception (air enema). POCUS was performed in patients who presented with any of the following symptoms: abdominal pain, irritability, bloody stool, abdominal mass or distension, vomiting, lethargy, diarrhea, and fever. We excluded patients transferred from another hospital or outpatient department with a suspected or confirmed diagnosis of intussusception as well as patients aged >6 years.

### Outcome Measures

**Figure 1:**
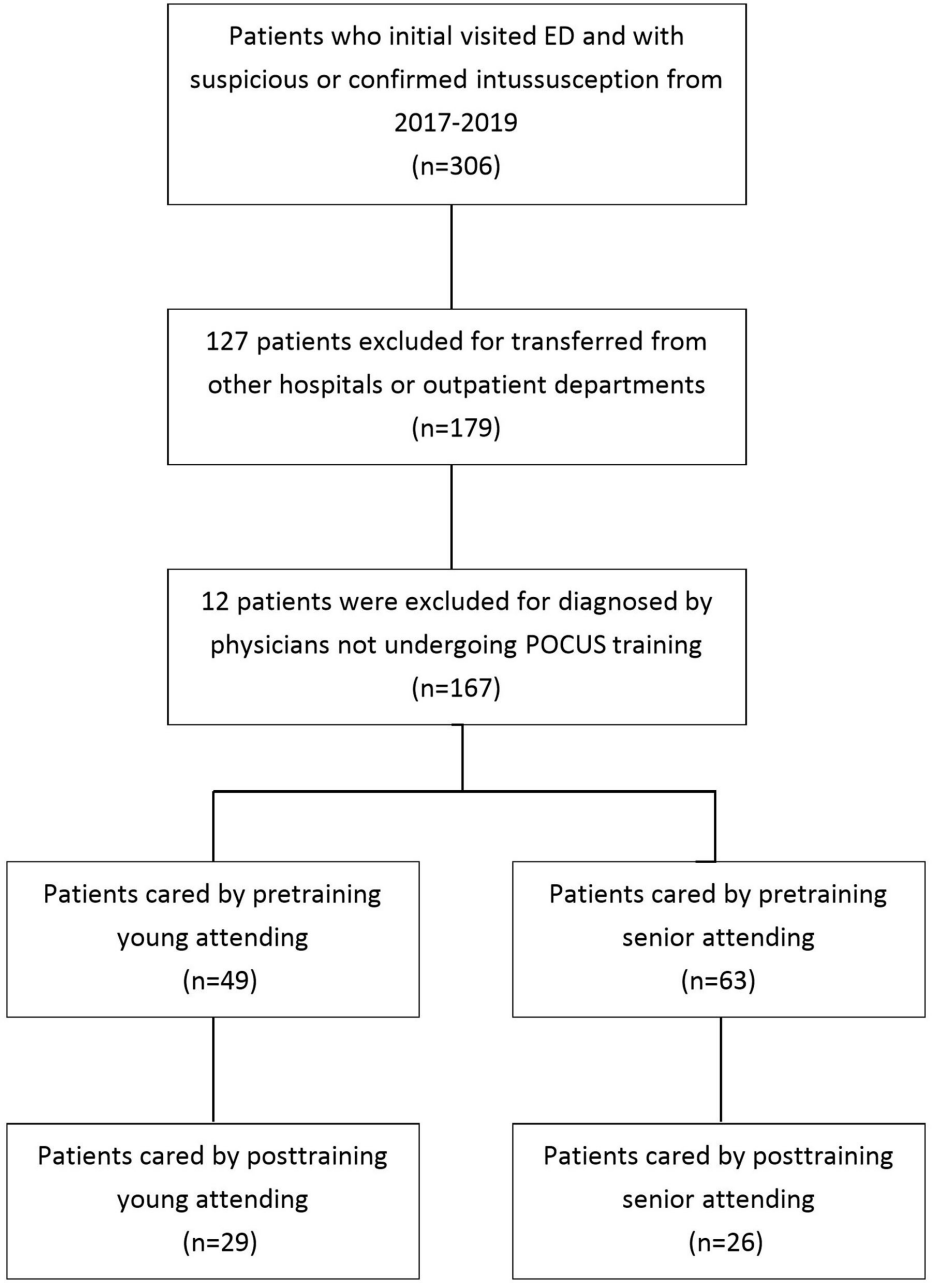
Study flowchart.

POCUS scans were categorized as “negative” or “suspected” for intussusception. A negative result on POCUS was defined as no obvious target or pseudokidney sign. “Misdiagnosis” was defined when a patient received a confirmed diagnosis of intussusception on an ED revisit, admission due to the lack of a tentative diagnosis of intussusception (false negative) or negative finding of air reduction by clinical suspicion during the initial ED visit, or admission as a suspected case of POCUS (false positive). All POCUS procedures were performed using a Logiq P7 (GE Ultrasound) with a 4–12-MHz linear or 4-MHz curvilinear transducer by 1 of 12 pediatric emergency attending physicians. During the study period, 14 attending physicians served in the pediatric ED. Every attending physician had completed a 5-year pediatric medicine training program offered for residents trained at our hospital. These attending physicians were categorized into two groups according to seniority in the pediatric ED: junior (*n* = 6; <10 years of work experience, range: 1–6 years) and senior (*n* = 8; ≥10 years of work experience, range: 10–17 years). One senior attending physician who resigned and one new junior attending physician who had not received POCUS training were excluded from the analysis. The other 12 attending physicians had completed an 8-h pediatric US course certified by the Taiwan Society of Pediatric Emergency Medicine by the end of December 2018 (Figure 1). Four of the senior attending physicians had prior POCUS training in pediatric fellowships, including two neurologists and two gastroenterologists. Patients who had been evaluated by an attending physician before and after POCUS training were assigned to the pre-training and post-training groups, respectively. The separating timepoint between these groups was the end of December 2018, when the POCUS training protocol for intussusception was implemented. The primary outcome in our study was the misdiagnosis rate, and the secondary outcomes were the door-to-reduction time (interpretations and time from ED arrival to performing air reduction), length of hospital stays, reduction failure rate, and rate of US consultation with an expert gastroenterologist.

### Statistical Analysis

We conducted a power analysis using G Power 3.1 to evaluate the minimal sample size required using a two-tailed test, a large effect size (*d* = 0.73, calculated by obtained groups' mean and standard deviation), and an alpha of 0.05. Result showed that a total sample of 100 patients with 50 patients in each group was required to achieve a power of 0.80. Thus, we achieved the minimal required sample size to test the appreciable difference between two independent group means. We also performed a power analysis to determine an appreciable difference in the outcome parameters in addition to the *p*-value. We collected patients' demographic data, including sex, age, and length of stay in the pediatric ED. Using an electronic record database, we recorded the number of patients visiting the ED, the number of revisits within 72 h, diagnoses, and management (including door-to-reduction time, reduction failure rate, and US consultation rate). Data were compared between the pre-training and post-training groups and analyzed in SPSS for Windows (version 22.0; SPSS, Chicago, IL, USA). The χ^2^ test was used to assess categorical data, and a two-tailed *t*-test was used to assess continuous variables. *P* < 0.05 was considered statistically significant.

## Results

Of the 306 enrolled children, 127 patients were excluded because they were transferred from other hospitals or outpatient departments for further management. An additional 12 patients were excluded because they had received diagnoses by physicians who did not undergo POCUS training. Finally, 167 patients were included in the analysis, of whom 163 (97.6%) received air enema, and the remaining 4 did not receive air enema for diagnosis or treatment. Of the four patients who did not receive enema, two received US from an expert and exhibited intussusception with mass lesions, including one patient with a diagnosis of lymphoma and intussusception and one with ileo-ileo-colic intussusception with an ischemic terminal ileum on operative findings. These two patients went directly to the operation room; the other two “suspected cases” then got an ultrasound from the expert and found no intussusception. Of the 163 patients receiving air enema, 130 (79.7%) were confirmed to have intussusception and 49 (30.3%) also received US from an expert before the enema. All the 167 patients were enrolled by 20 included attending physicians (Figure 1). Of them, 130 were confirmed to have intussusception through air enema. Patient characteristics are listed in Table 1.

**Table 1:** Comparison of clinical characteristics and laboratory values between the PRE and POST groups.

	**PRE**	**POST**	***P***
	**(*n* = 112)**	**(*n* = 55)**	
Age (Y)	2.0 ± 1.5	1.8 ± 1.7	0.385
Male sex	65/112	34/55	0.640
Duration of symptoms (D)	1.8 ± 3.0	1.9 ± 4.4	0.795
Pain/irritability	90/112	46/55	0.608
Vomiting	65/112	33/55	0.706
Fever	20/112	10/55	0.822
Bloody stool	20/112	6/55	0.244
Diarrhea	14/112	10/55	0.325
WBC, thousand/μL	11,325 ± 4,357	11,016 ± 2,976	0.604
CRP, mg/L	9.3 ± 13.8	13.9 ±	0.314

The pre-training and post-training groups included 112 and 55 patients, respectively. Abdominal pain or irritability (80.9%) was the most common symptom, followed by vomiting (58.3%), fever (17.8%), bloody stool (15.4%), and diarrhea (14.2%). The clinical features did not significantly differ between the two groups. Table 2 lists the outcomes of patients and performance characteristics of physicians in the pre-training and post-training groups. In the post-training group, 45 patients received an enema as the first choice, 7 of whom obtained negative findings on enema. Another 10 patients received US from an expert for confirmation of diagnosis and consequently received an enema for treatment. The rate of misdiagnosis of patients who did not receive expert US consultation in the post-training group was 15.5% (7/45). Compared with the pre-training group, the post-training group had a significantly lower misdiagnosis rate (12.7 vs. 43.7%, *P* < 0.001), shorter door-to-reduction time (118.2 ± 124.5 min vs. 198 ± 250.2 mis, *P* = 0.006), and lower rate of US consultation (20 vs. 41%, *P* = 0.007). Here, the power of misdiagnosis rate, door-to-reduction time, and US consultation was 0.98, 0.69, and 0.77, respectively.

**Table 2:** Comparison of misdiagnosis and outcome between the PRE and POST group.

	**PRE**	**POST**	***P***
Misdiagnosis	49/112 (43.7%)	7/55 (12.7%)	<0.001
Door-to-reduction time (min)	198.8 ± 250.2	118.2 ± 124.5	0.006
Hospital stay (D)	4.5 ± 2.0	3.9 ± 2.0	0.128
Reduction failure	15/112 (13.3%)	7/55 (12.7%)	0.921
US consultation	46/112 (41%)	11/55 (20%)	0.007

Table 3 compares misdiagnosis-associated clinical symptoms between junior and senior physicians. In total, 12 (7 senior and 5 junior) pediatric attending physicians received POCUS training. In both the pre-training and post-training periods, the misdiagnosis rate was significantly higher in the junior physicians than in the senior physicians. Both junior and senior physicians made fewer misdiagnoses in the post-training period than in the pre-training period (59.1 vs. 25.9%, *P* = 0.003; 31.7 vs. 0%, *P* < 0.001). The misdiagnosis rate for patients presenting with pain or vomiting was significantly lower in the post-training period than in the pre-training period (37.7 vs 13%, *P* = 0.003, and 41.5 vs. 6%, *P* < 0.001, respectively). Diarrhea and fever were the most common presentations leading to misdiagnosis at both pre-training and post-training.

**Table 3:** Comparison of misdiagnosis associated clinical symptoms and seniority of attending (VS) between the PRE and POST groups.

	**PRE**	**POST**	***P***
**Symptoms**			
Diarrhea	6/14 (42.8%)	4/10 (40%)	1.000
Fever	11/22 (50%)	4/10 (40%)	0.712
Pain	34/90 (37.7%)	6/46 (13%)	0.003
Blood stool	7/20 (35%)	0/6 (0%)	0.146
Vomiting	27/65 (41.5%)	2/33 (6%)	<0.001
**Seniority**			
Young VS	29/49 (59.1%)	7/29 (25.9%)	0.003
Senior VS	20/63 (31.7%)	0/26 (0%)	<0.001

## Discussion

To our knowledge, this is the first observational cohort study to assess the effect of POCUS training on the misdiagnosis of pediatric intussusception. To achieve this objective, we performed a comparison of the diagnostic performance between junior and senior attending physicians. We found several crucial findings. First, intussusception misdiagnosis rate significantly decreased, with both shorter time to air reduction and decreased rate of US consultations with an expert, after the pediatric emergency physicians underwent a single, focused training session. Second, although both the junior and senior attending physicians had minimal experience in US, they performed US with a high diagnostic accuracy after the brief training. Furthermore, diarrhea or fever as presenting symptoms of intussusception often led junior attending physicians to misdiagnosis intussusception as gastroenteritis even after POCUS training.

Patients with intussusception often present with non-specific abdominal complaints that may mimic other diseases, possibly increasing diagnostic error risk (26, 27). Studies have reported that up to 60% of intussusception cases are initially misdiagnosed (28). In our study, the misdiagnosis rate was 43% before POCUS training. Our study results proved that intussusception poses a diagnostic challenge for emergency physicians, even at medical centers. Furthermore, our data demonstrate that inexperienced physicians can master the use of POCUS for diagnosing intussusception and that ~70% of misdiagnoses are might be preventable after POCUS training. This result corroborates that of previous studies that emergency physicians can make accurate diagnoses after brief training sessions (20, 25). In our study, the training time was 8 h. Similar performance characteristics were reported in a study on emergency physicians who received a minimum of 1 h of instruction (25). Our current results indicated that junior attending physicians performed US screening for suspected intussusception as effectively as senior attending physicians after training. This observation may be attributed to the lower threshold of searching for intussusception afforded by POCUS (18).

Although POCUS is a reliable tool for the diagnosis of intussusception in children, an initial clinical suspicion is needed to convince physicians to use the test. Another finding in our study was that the presence of diarrhea or fever in patients ultimately diagnosed with intussusception may have led to misdiagnosis as acute gastroenteritis. Previous studies have reported that bacterial enteritis significantly increases the relative risk of intussusception (29, 30). An increased risk of intussusception was found after *Salmonella, E. coli, Shigella*, and *Campylobacter* infection (29). In the present study, 15% of the evaluated children had concurrent fever. A previous study reported fever in 40% of the intussusception cases (31). Viruses commonly cause intussusception in young children (4). A strong association between intussusception and the recent adenovirus infection has been reported in various populations, which may lead to the presentation of viral illness, including symptoms of fever or diarrhea, before the onset of intussusception (32). Our study revealed that the presence of abdominal pain with fever and diarrhea led to misdiagnosis of intussusception as gastroenteritis by a junior attending physician even after focused training. Therefore, physicians must apply clinical judgment in making decisions to use the POCUS. Our study findings may help physicians to maintain a high level of suspicion when they encounter a history of diarrhea accompanied by abdominal pain and fever in infants and children. These signs should not be immediately diagnosed as gastroenteritis; rather, and the relevant patients should be examined thoroughly with a high degree of suspicion for intussusception.

Any misdiagnosis may lead to delays in intervention and treatment. In our study, the post-training group had a decreased door-to-reduction time and lower US consultation percentage. Previous studies have reported that crowding could also lead to misdiagnosis (33). POCUS may help make a more timely diagnosis and have the potential to facilitate radiology or surgical department reduction procedures. Furthermore, ED patients with pediatric US capability would also benefit to efficiently improve resource use for patients with suspected intussusception.

Several limitations of the present study must be noted. First, this was a retrospective study, potential bias may have occurred in the analysis; the numbers of discharged patients with negative POCUS result were not totally recorded; the symptoms and signs were obtained from medical records, and thus, some symptoms or signs may have been overlooked. Moreover, our single-center results may not be generalizable to other similar acute care settings; it might also be relevant that all our attending physicians were pediatricians. Finally, in some cases, intussusception may first be present and then may spontaneously decrease before interventions such as air enema, which may have contributed to misdiagnosis (false positive).

In summary, the use of POCUS for intussusception screening could help reduce misdiagnosis rates, shorten observation times, and lessen the need for referrals for expert US. The evidence obtained in this study suggests that the challenge of misdiagnosis is not easily amenable for junior physicians when diarrhea or fever is present. Combining clinical judgment and POCUS remains the mainstay in the evaluation of children with a suspected diagnosis of intussusception. Future prospective studies on the optimal training duration and content are warranted.

## Data Availability Statement

The raw data supporting the conclusions of this article will be made available by the authors, without undue reservation.

## Author Contributions

Y-JC: conceptualization. C-LC and S-YC: methodology and software. H-JH: validation and writing. C-CL and Y-CH: formal analysis. C-JW and S-YY: investigation. W-CL and P-WW: resources and data curation. All authors listed have made a substantial, direct and intellectual contribution to the work, and approved it for publication.

## Conflict of Interest

The authors declare that the research was conducted in the absence of any commercial or financial relationships that could be construed as a potential conflict of interest.
